# Consumption of ultra-processed foods and health status: a systematic review and meta-analysis

**DOI:** 10.1017/S0007114520002688

**Published:** 2021-02-14

**Authors:** G. Pagliai, M. Dinu, M. P. Madarena, M. Bonaccio, L. Iacoviello, F. Sofi

**Affiliations:** 1 Department of Experimental and Clinical Medicine, University of Florence, 50134 Florence, Italy; 2 Unit of Clinical Nutrition, Careggi University Hospital, 50134 Florence, Italy; 3 Department of Epidemiology and Prevention, IRCCS Neuromed, Pozzilli, 86077 Isernia, Italy; 4 Department of Medicine and Surgery, Research Center in Epidemiology and Preventive Medicine (EPIMED), University of Insubria, 21100 Varese, Italy

**Keywords:** Ultra-processed food, Health, CVD, Mortality, Meta-analysis

## Abstract

Increasing evidence suggests that high consumption of ultra-processed foods (UPF) is associated with an increase in non-communicable diseases, overweight and obesity. The present study systematically reviewed all observational studies that investigated the association between UPF consumption and health status. A comprehensive search of MEDLINE, Embase, Scopus, Web of Science and Google Scholar was conducted, and reference lists of included articles were checked. Only cross-sectional and prospective cohort studies were included. At the end of the selection process, twenty-three studies (ten cross-sectional and thirteen prospective cohort studies) were included in the systematic review. As regards the cross-sectional studies, the highest UPF consumption was associated with a significant increase in the risk of overweight/obesity (+39 %), high waist circumference (+39 %), low HDL-cholesterol levels (+102 %) and the metabolic syndrome (+79 %), while no significant associations with hypertension, hyperglycaemia or hypertriacylglycerolaemia were observed. For prospective cohort studies evaluating a total population of 183 491 participants followed for a period ranging from 3·5 to 19 years, highest UPF consumption was found to be associated with increased risk of all-cause mortality in five studies (risk ratio (RR) 1·25, 95 % CI 1·14, 1·37; *P* < 0·00001), increased risk of CVD in three studies (RR 1·29, 95 % CI 1·12, 1·48; *P* = 0·0003), cerebrovascular disease in two studies (RR 1·34, 95 % CI 1·07, 1·68; *P* = 0·01) and depression in two studies (RR 1·20, 95 % CI 1·03, 1·40; *P* = 0·02). In conclusion, increased UPF consumption was associated, although in a limited number of studies, with a worse cardiometabolic risk profile and a higher risk of CVD, cerebrovascular disease, depression and all-cause mortality.

Ultra-processed foods (UPF) are, according to the *NOVA* classification, ‘formulations of ingredients, mostly for industrial use only, derived from a series of industrial processes’^(1)^. Examples of UPF are breakfast cereals, savoury snacks, reconstituted meat products, frankfurters, pre-packaged frozen dishes, soft and/or sweetened drinks, distilled alcoholic beverages and supplements.

UPF represents an important and growing part of the world’s food supply. Recent studies have reported that these foods account for a significant percentage of about 50–60 % of the energy content in the usual diet of the average US, Canadian or British consumer^(2–4)^. The increase in the volume of industrially processed products in the global food supply has coincided with an increasing prevalence of obesity and non-communicable diseases in many countries^(5)^, suggesting a possible association between UPF consumption and obesity risk, but studies on the potential health effects of UPF are limited.

Some cross-sectional studies have reported a significant association between UPF consumption, obesity^(6–9)^ and the metabolic syndrome^(10)^, while others have shown no association^(11,12)^. In addition, results from a large French prospective cohort study, the NutriNet-Santé study, found that high UPF consumption led to a significant increase in the risk of CVD^(13)^, diabetes^(14)^, depressive symptoms^(15)^ and cancer^(16)^. To date, despite great interest in the subject in both scientific and lay communities, there is a lack of consensus in terms of evaluation and impact of UPF on health and no systematic reviews, and meta-analyses were conducted so far on adults. Our study aimed to assess the relationship between UPF consumption as defined by *NOVA* and health status by conducting a comprehensive systematic review with meta-analysis of all the cross-sectional and cohort studies published so far.

## Methods

### Search strategy and selection of studies

The study was conducted following the Preferred Reporting Items for Systematic Reviews and Meta-analysis guidelines^(17)^. The protocol was registered at www.crd.york.ac./PROSPERO/uk as CRD42020165495. Two authors (G. P. and M. D.) independently performed systematic literature searches in MEDLINE, Embase, Scopus, Web of Science and Google Scholar databases, from inception to June 2020. Further studies were searched by checking the references of the identified articles. The keywords ‘ultra processed’ or ‘ultraprocessed’ or ‘ultra-processed’ or ‘NOVA’, and ‘food’ or ‘foods’ were used in combination as medical subject heading (MeSH) terms and text words. No language limitations were applied. Missing data or additional information were requested from the corresponding authors of the articles.

Two investigators (G. P. and M. D.) independently assessed articles potentially relevant for eligibility. Observational studies that reported a measure of association (risk estimates with CI or standard errors or sufficient data to calculate them) between the UPF consumption – defined by the *NOVA* Food Classification System^(18,19)^ and evaluated by dietary recalls, food records or questionnaires – and health indicators were considered eligible for inclusion. The decision to include the studies was based on the study title, abstract and full-text screening. The inclusion and exclusion criteria are summarised in online Supplementary Table S1, following the PECOS (Population, Exposure, Comparison, Outcome, Study) design format. Eligible studies were included if they met the inclusion criteria for the study population (clinically healthy subjects aged ≥18 years), exposure (high UPF consumption), reference group (low UPF consumption), outcome (any health indicator), study design (cross-sectional and prospective cohort studies) and statistics (sufficient data to allow calculation of differences between subjects consuming high UPF levels and those consuming low UPF levels). Case–control studies were excluded to minimise bias in recall and selection. Review articles, letters to the editor, comments, case reports and randomised controlled trials were also excluded. Discrepancies were resolved through consensus and discussion with a third investigator (M. P. M.) if consensus could not be reached.

### Data extraction

Data extraction was carried out in duplicate by two investigators (M. D. and G. P.) using a standardised form. Disagreements were resolved by consensus or by a third investigator (M. P. M.) if consensus could not be reached. The following data were extracted from the original articles: main author, year of publication, country of study population, cohort, number of participants evaluated and events, length of follow-up (years), age of the population at baseline, sex, definition of outcome of interest, method used to assess UPF intake, comparison, measures of effect size and CI, and details of adjustment for confounding factors in the multivariate model. If the results were reported separately for men and women, they were included in the analysis as separate cohorts.

### Assessment of methodological quality

Two investigators (G. P. and M. D.) independently assessed the methodological quality of each included study using the National Institutes of Health study quality assessment tool for observational cohort and cross-sectional studies^(20)^. This tool has fourteen items in total, with an overall rating based on weaknesses in critical domains (see online Supplementary Table S2). The critical domains were the following: research question, exposure assessed prior to outcome measurement, exposure measurements and evaluation, outcome measurements, and statistical analysis. The final results lead to an overall methodological evaluation of good, fair or poor. Disagreements were resolved by consensus or by a third investigator (M. P. M.) if consensus could not be reached.

### Statistical analysis

All data were analysed using Review Manager (RevMan; version 5.3 for Macintosh). A random-effects model (DerSimonian and Laird method) was applied to combine multivariable-adjusted risk ratios (RR) or OR of the highest *v*. the lowest category of UPF consumption. The pooled results were reported as RR and were presented with 95 % CI with two-sided *P* values. Meta-analysis was conducted if ≥2 studies were available for an outcome. Outcomes expressed in *β*-coefficient or prevalence ratio have been excluded from the meta-analysis. The statistical heterogeneity between studies was estimated using the *χ* ^2^ Cochran’s Q-test with the *I* ^2^ statistic, which provides an estimate of the amount of variance between studies due to the heterogeneity rather than sampling error^(21)^. Where *I* ^2^ exceeded 50 %, heterogeneity was considered substantial and subgroup analyses were performed to explore the source of the heterogeneity^(22)^. The robustness of the results was established by eliminating each study one by one from the meta-analysis and recalculating the summary estimate (the ‘leave one out’ approach). If ≥5 studies were available, the possibility of publication bias was explored by visual inspection of funnel plot of the effect size against standard error. A *P* <0·05 was considered statistically significant.

## Results

### Search results

**Fig. 1.**
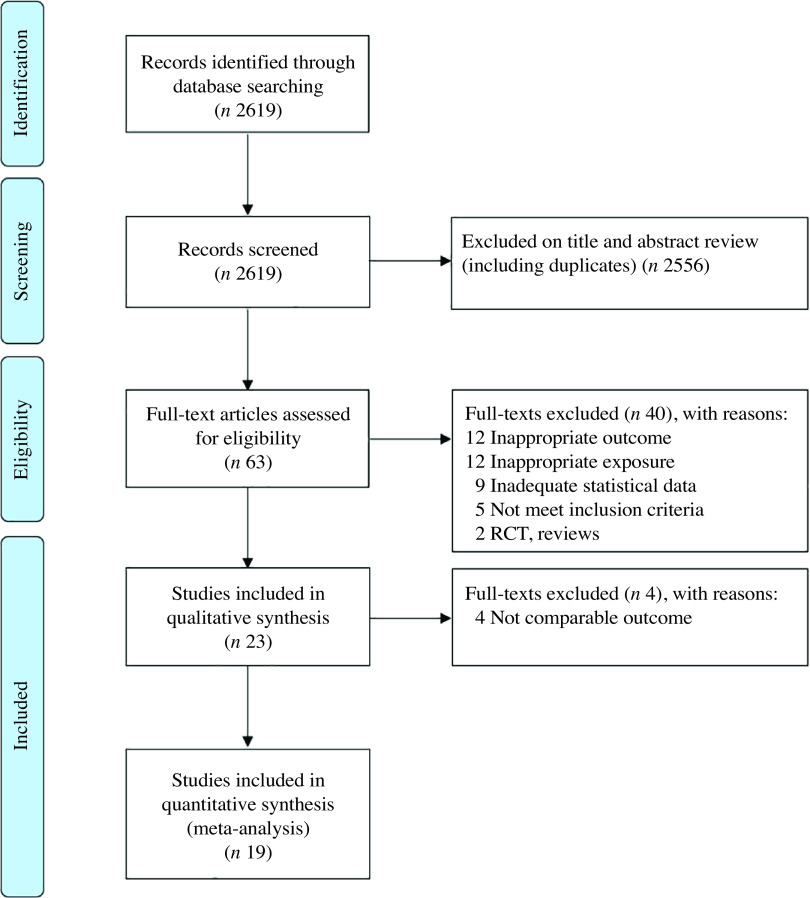
Preferred Reporting Items for Systematic Reviews and Meta-analysis flow diagram for search strategy. RCT, randomised controlled trials.

The selection process is shown in Fig. 1, according to the Preferred Reporting Items for Systematic Reviews and Meta-analysis guidelines. The search produced 2619 articles. After title and abstract screening, sixty-three articles were selected for the evaluation of the full text. At the end of the selection process, twenty-three articles were included in the qualitative analysis and nineteen in the quantitative analysis.

Selected cross-sectional studies (*n* 10 studies) examined the association between the UPF consumption and the following health outcomes: overweight/obesity (*n* 5), high waist circumference or abdominal obesity (*n* 5), BMI gain (*n* 3), hypertension (*n* 3), low HDL-cholesterol (*n* 3), the metabolic syndrome (*n* 3), hypertriacylglycerolaemia (*n* 3), hyperglycaemia (*n* 3), waist circumference gain (*n* 2), irritable bowel syndrome (*n* 1), and C-reactive protein levels (*n* 1). Selected prospective cohort studies (*n* 13) examined the association between the UPF consumption and all-cause mortality (*n* 5), CVD risk/mortality (*n* 3), overweight/obesity (*n* 2), depression (*n* 2), IHD/cerebrovascular risk/mortality (*n* 2), waist circumference gain (*n* 1), hypertension (*n* 1), frailty (*n* 1), CHD (*n* 1), overall cancer risk/mortality (*n* 1), breast cancer (*n* 1), prostate cancer (*n* 1) and colorectal cancer (*n* 1).

### Cross-sectional studies

Table 1 presents the main characteristics of the ten cross-sectional studies included in the systematic review. The overall analysis comprised 113 753 participants. Three studies were conducted in Brazil^(6,8,23)^, two in Canada^(9,24)^, two in the USA^(7,10)^, one in France^(25)^, one in the UK^(26)^ and one in Lebanon^(12)^. The evaluation of UPF consumption was conducted through 24-h dietary recall in five studies^(7,9,10,24,25)^, 24-h food records in one study^(6)^, 4-d food records in one study^(26)^ and FFQ in the remaining three studies^(8,12,23)^. Exposure was assessed through the total energy contribution from UPF. The methodological quality score was fair in six studies^(6–8,10,25,26)^ and poor in four studies^(9,12,23,24)^.

**Table 1.** Characteristics of cross-sectional studies evaluating ultra-processed food (UPF) consumption and different health outcomes

Author (year)	Country (cohort)	Subjects (*n*)	Age (years)	Sex	Outcome	Assessment of UPF intake	Comparison	Exposure	Reference	OR	95 % CI	Adjustment	Study quality
Louzada *et al*. (2015)^(6)^	Brazil (National Household Budget Survey)	12 586	20–39	M/F	Obesity	24-h food records	Q5 *v*. Q1	≥44 % of TE	≤13 % of TE	1·35	0·83, 2·18	Age, sex, race, region, urban status, smoking status, physical activity, quintiles of years of education per capita household income, consumption of fruits, vegetables and beans, and interaction between sex and income	Fair
		7534	40–59							1·19	0·92, 1·55		
		2589	≥60							1·55	0·58, 4·12		
Juul *et al*. (2018)^(7)^	USA (NHANES)	15 977	20–64	M/F	Overweight/obesity	24-h dietary recall	Q5 *v*. Q1	≥74·2 % of TE	≤36·5 % of TE	1·48	1·25, 1·76	Age, sex, educational attainment, race/ethnicity, family income, marital status, smoking status and physical activity	Fair
					BMI gain					1·61*	1·11, 2·10		
					WC gain					4·07*	2·94, 5·19		
					High WC					1·62	1·39, 1·89		
Lavigne-Robichaud *et al*. (2018)^(24)^	Canada (Aschii Environment and Health Study)	811	≥18	M/F	Metabolic syndrome	24-h dietary recall	Q5 *v*. Q1	83 % of TE	21·1 % of TE	1·90	1·14, 3·17	Age, sex, area of residence, current smoker, alcohol drinker and total dietary energy intake	Poor
					Hypertension					0·99	0·59, 1·68		
					Hyperglycaemia					1·76	1·04, 2·97		
					Low HDL-cholesterol					2·05	1·25, 3·38		
					Hypertriacylglycerolaemia					0·93	0·57, 1·52		
					High WC					1·18	0·33, 4·32		
Nasreddine *et al*. (2018)^(12)^	Lebanon	302	≥18	M/F	Metabolic syndrome	80-item FFQ	Q4-Q2 *v*. Q1	NA	NA	1·11	0·26, 4·65	Age, sex, marital status, area of residence, level of education, income, smoking status, physical activity, total energy intake and BMI	Poor
					Hypertension					3·10	0·58, 16·66		
					Hyperglycaemia					0·52	0·15, 1·83		
					Low HDL-cholesterol					1·82	0·52, 6·42		
					Hypertriacylglycerolaemia					1·08	0·28, 4·11		
Schnabel *et al*. (2018)^(25)^	France (NutriNet-Santé)	33 343	50·4	M/F	IBS	24-h dietary recall	Q4 *v*. Q1	>20·6 % of TE	<9·7 % of TE	1·25	1·12, 1·39	Age, sex, income level, education level, marital status, residence, BMI, physical activity, smoking status, energy intake, season of food records, time between food record and FGID questionnaire, and mPNNS-GS	Fair
Silva *et al*. (2018)^(8)^	Brazil (ELSA-Brazil)	8977	35–64	M/F	Obesity	114-item FFQ	Q4 *v*. Q1	>29 % of TE	<16 % of TE	1·43	1·20, 1·72	Age, sex, race/skin colour, per capita family income, physical activity, smoking, hypertension, diabetes and energy intake	Fair
					Overweight					1·32	1·15, 1·53		
					BMI gain					0·80*	0·53, 1·07		
					High WC					1·21	1·01, 1·46		
Lopes *et al*. (2019)^(23)^	Brazil (ELSA-Brazil)	15 105	35–74	M	CRP	114-item FFQ	Q3 *v*. Q1	NA	NA	0·93	0·84, 1·02	Age, race/skin colour, educational attainment, smoking, physical activity and BMI	Poor
				F						1·00	0·92, 1·08		
Martínez Steele *et al*. (2019)^(10)^	USA (NHANES)	6385	≥20	M/F	Metabolic syndrome	24-h dietary recall	Q5 *v*. Q1	>71 % of TE	<40 % of TE	1·28†	1·09, 1·50	Age, sex, race/ethnicity, ratio of family income to poverty, educational attainment, smoking status and physical activity	Fair
					Hypertension					1·19†	1·03, 1·38		
					Hyperglycaemia					1·06†	0·93, 1·19		
					Low HDL-cholesterol					1·34†	1·19, 1·49		
					Hypertriacylglycerolaemia					1·12†	0·97, 1·28		
					High WC					1·26†	1·13, 1·39		
Nardocci *et al*. (2019)^(9)^	Canada (CCHS)	19 363	≥18	M/F	Obesity	24-h dietary recall	Q5 *v*. Q1	75·95 % of TE	20·08 % of TE	1·32	1·05, 1·57	Age, sex, education, income, physical activity, smoking status, immigrant status, zone of residence, reporting group and measurement type	Poor
Rauber *et al*. (2020)^(26)^	UK. (NDNS)	6143	≥19	M/F	Obesity	4-d food records	Q4 *v*. Q1	>73·1 % (F) or >76·2 % (M) of TE	<35·2 % (F) or <36·3 % (M) of TE	1·90	1·39, 2·61	Age, sex, ethnicity, region, social class, survey year, physical activity, smoking status, sleep duration and following a diet to lose weight.	Fair
					BMI gain					1·66*	0·96, 2·36		
					WC gain					3·56*	1·79, 5·33		
					High WC					1·34	1·00, 1·79		

CCHS, Canadian Community Health Survey; CRP, C-reactive protein; E, energy; ELSA, Brazilian Longitudinal Study of Adult Health; F, female; FGID, four functional gastrointestinal disorders; IBS, irritable bowel syndrome; NHANES, National Health and Nutrition Examination Survey; M, male; mPNNS-GS, Modified Programme National Nutrition Santé Guideline Score; NDNS, UK National Diet and Nutrition Survey; TE, total energy; WC, waist circumference.

* Values are expressed as *β*-coefficients.

† Values are expressed as prevalence ratios.

**Fig. 2.**
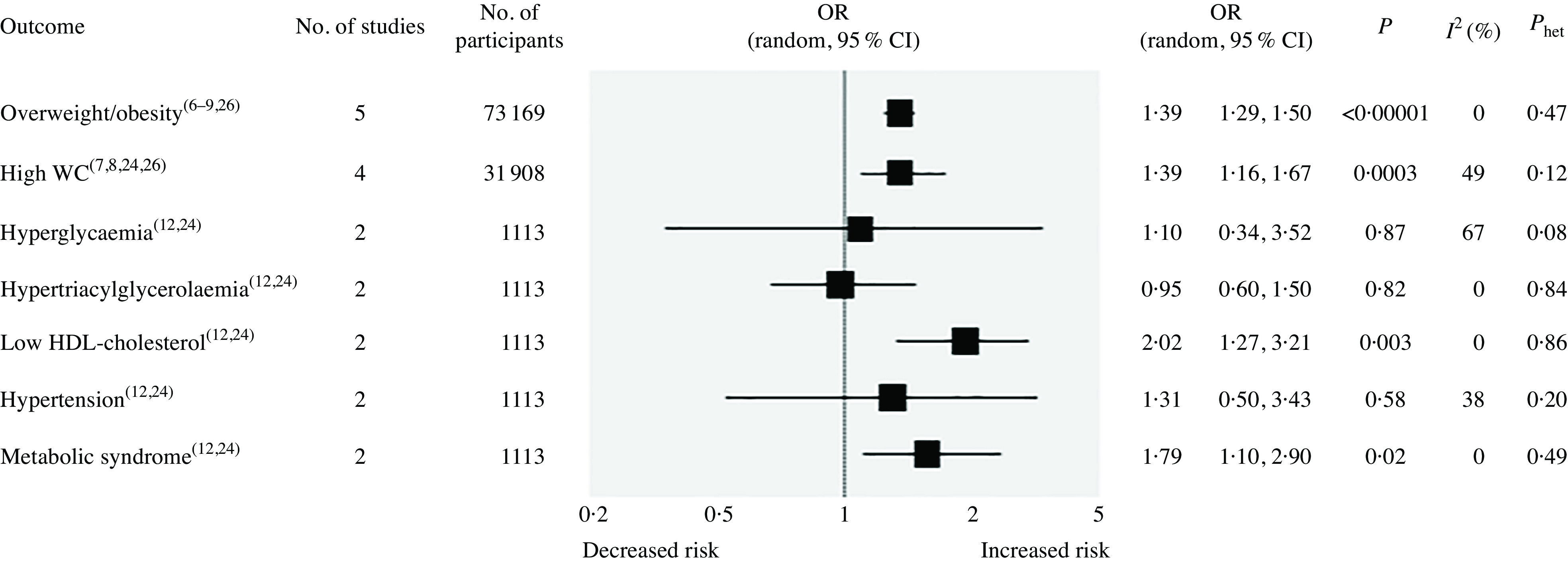
Forest plot of cross-sectional studies investigating the association between ultra-processed foods consumption and different health outcomes. *P* value is for *Z* test of no overall association between exposure and outcome; *P*_het_ is for test of no differences in association measure among studies; *I* ^2^ estimates from heterogeneity rather than sampling error. WC, waist circumference.

Meta-analytic pooling under a random-effects model indicated a significant association between the highest UPF consumption and increased risk of overweight/obesity in five studies^(6–9,26)^ with a total population of 73 169 subjects (OR 1·39, 95 % CI 1·29, 1·50; *P* < 0·00001), without any evidence of statistical heterogeneity between studies (*I* ^2^ = 0 %; *P* = 0·47) (Fig. 2). Similarly, a statistically significant association was found between highest UPF consumption and increased risk of high waist circumference or abdominal obesity in four studies^(7,8,24,26)^ with a population of 31 908 (OR 1·39, 95 % CI 1·16, 1·67; *P* = 0·0003), without any statistical heterogeneity between studies (*I* ^2^ = 49 %; *P* = 0·12). In addition, highest UPF intake was associated with increased risk of the metabolic syndrome (OR 1·79, 95 % CI 1·10, 2·90; *P* = 0·02) and reduced HDL-cholesterol levels (OR 2·02, 95 % CI 1·27, 3·21; *P* = 0·003), with no evidence of statistical heterogeneity between studies (*I* ^2^ = 0 %; *P* = 0·49 and *I* ^2^ = 0 %; *P* = 0·86, respectively) in two studies^(12,24)^ involving a limited population of 1113 subjects. On the other hand, no statistically significant associations emerged between highest consumption of UPF and hypertension^(12,24)^ (OR 1·31, 95 % CI 0·50, 3·43; *P* = 0·58), hyperglycaemia^(12,24)^ (OR 1·10, 95 % CI 0·34, 3·52; *P* = 0·87) or hypertriacylglycerolaemia^(12,24)^ (OR 0·95, 95 % CI 0·60, 1·50; *P* = 0·82).

### Prospective cohort studies

Table 2 presents the main characteristics of the thirteen prospective cohort studies included in the systematic review. The overall analysis comprised 183 491 participants followed over a period ranging from 3·5 to 19 years. Two cohorts were based in Spain^(27–32)^, one in France^(13,15,16,33)^, one in Brazil^(34)^, one in Italy^(35)^ and one in the USA^(36)^. The evaluation of UPF consumption was conducted through 24-h dietary recalls, FFQ and dietary history. Exposure is extremely variable and ranges from the contribution of the total energy of UPF to servings per d or daily intake. The methodological quality score was good in all studies but one^(32)^.

**Table 2.** Characteristics of prospective cohort studies evaluating ultra-processed food (UPF) consumption and different health outcomes

Author (year)	Country (cohort)	*n*/*N*	Follow-up (years)	Age (years)	Sex	Outcome	Assessment of UPF intake	Comparison	Exposure	Reference	RR	95 % CI	Adjustment	Study quality
Mendonça *et al*. (2016)^(27)^	Spain (SUN)	1939/8451	8·9	37·6	M/F	Overweight/obesity	136-item FFQ	Q4 *v*. Q1	6·1 servings/d	1·5 servings/d	1·26	1·10, 1·45	Age, sex, marital status, educational status, physical activity, TV watching, siesta sleep, smoking status, snacking between meals, following a special diet at baseline, baseline BMI and consumption of fruits and vegetables	Good
Mendonça *et al*. (2017)^(28)^	Spain (SUN)	1702/14 790	9·1	≥18	M/F	Hypertension	136-item FFQ	T3 *v*. T1	5·0 servings/d	2·1 servings/d	1·21	1·06, 1·37	Age, sex, physical activity, hours of TV watching, baseline BMI, smoking status, use of analgesics, following a special diet at baseline, family history of hypertension, hypercholesterolaemia, alcohol consumption, total energy intake, olive oil intake and consumption of fruits and vegetables	Good
Fiolet *et al*. (2018)^(16)^	France (NutriNet-Santé)	2228/104 980	5·4	42·8	M/F	Overall cancer risk	24-h dietary recall	Q4 *v*. Q1	≥23·3 % of TE	≤11·8 % of TE	1·23	1·08, 1·40	Age, sex, energy intake without alcohol, number of 24-h dietary records, smoking status, educational level, physical activity, height, BMI, alcohol intake, family history of cancers, intakes of lipids, sodium, and carbohydrates, Western dietary pattern, menopausal status, hormonal treatment for menopause, oral contraception and number of children	Good
		739/104 980				Breast cancer risk					1·13	0·89, 1·42		
		281/104 980				Prostate cancer risk					0·93	0·61, 1·40		
		153/104 980				Colorectal cancer risk					1·23	1·08, 1·40		
Adjibade *et al*. (2019)^(15)^	France (NutriNet-Santé)	2221/26 730	5·4	18–86	M/F	Depressive symptoms	24-h dietary recall	Q4 *v*. Q1	19–76 % of TE	0–10 % of TE	1·13	1·00, 1·28	Age, sex, BMI, marital status, educational level, occupational categories, household income per consumption unit, residential area, number of 24-h dietary records, inclusion month, energy intake without alcohol, alcohol intake, smoking status, physical activity, use of antidepressants during follow-up, baseline CES-D score and CDS score	Good
Blanco-Rojo *et al*. (2019)^(29)^	Spain (ENRICA)	44/11 898	7·7	46·9	M/F	All-cause mortality	Computer-based dietary history	Q4 *v*. Q1	42·83 % of TE	8·68 % of TE	1·44	1·01, 2·07	Age, sex, educational level, living alone, smoking status, former drinker, physical activity index, time of watching TV, time devoted to other sedentary activities, number of medications per d and specific chronic conditions diagnosed by a physician	Good
Canhada *et al*. (2019)^(34)^	Brazil (ELSA-Brazil)	972/11 827	3·8	35–74	M/F	Overweight/Obesity	114-item FFQ	Q4 *v*. Q1	30·84–73·84 % of TE	0–17·79 % of TE	1·20	1·03, 1·40	Age, sex, colour/race, centre, income, school achievement, smoking status, physical activity, baseline BMI and WC at baseline	Good
		1183/11 827				WC gain					1·33	1·12, 1·58		
Gómez-Donoso *et al*. (2020)^(30)^	Spain (SUN)	774/14 907	10·3	36·7	M/F	Depression	136-item FFQ	Q4 *v*. Q1	489 g/d	119 g/d	1·33	1·07, 1·64	Age, sex, year of entrance to the cohort, baseline BMI, total energy intake, physical activity, smoking status, marital status, living alone, employment status, working hours per week, health-related career, years of education, MedDiet Score, baseline self-perception of competitiveness, anxiety and dependence levels	Good
Kim *et al*. (2019)^(36)^	USA (NHANES III)	648/11 898	19	≥20	M/F	CVD mortality	81-item FFQ and 24-h dietary recall	Q4 *v*. Q1	5·2–29·8 times/d	0–2·6 times/d	1·13	0·74, 1·71	Age, sex, race/ethnicity, total energy intake, poverty level, education level, smoking status, physical activity, alcohol intake, BMI, hypertension status, total cholesterol and estimated glomerular filtration rate	Good
		2451/11 898				All-cause mortality					1·30	1·08, 1·57		
Rico-Campà *et al*. (2019)^(31)^	Spain (SUN)	355/19 899	10·4	37·6	M/F	All-cause mortality	136-item FFQ	Q4 *v*. Q1	>4 servings/d	<2 servings/d	1·62	1·13, 2·33	Age, sex, marital status, baseline BMI, total energy intake, smoking status, family history of CVD, alcohol consumption, CVD, cancer, diabetes, depression, hypertension, educational level, self-reported hypercholesterolaemia, snacking, following a special diet at baseline, physical activity and smoking status	Good
Sandoval-Insausti *et al*. (2020)^(32)^	Spain (Seniors-ENRICA)	132/1822	3·5	≥60	M/F	Incident frailty	Computer-based dietary history	Q4 *v*. Q1	34·9 % of TE	6·5 % of TE	3·67	2·00, 6·73	Age, sex, level of education, marital status, tobacco consumption, former-drinker status, chronic respiratory disease, coronary disease, stroke, osteoarthritis/arthritis, cancer, depression requiring treatment and number of medications used	Fair
Schnabel *et al*. (2019)^(33)^	France (NutriNet-Santé)	602/44 551	7·1	≥45	M/F	All-cause mortality	24-h dietary recall	Q4 *v*. Q1	>18·0 % of TE	<9·3 % of TE	1·25	0·99, 1·57	Age, sex, income level, education level, marital status, residence, BMI, physical activity, smoking status, energy intake, alcohol intake, season of food records, first-degree family history of cancer or CVD, number of food records and mPNNS-GS	Good
Srour *et al*. (2019)^(13)^	France (NutriNet-Santé)	1409/105 159	5·2	≥18	M/F	CVD risk	24-h dietary recall	Q4 *v*. Q1	>22 % of TE	<11 % of TE	1·23	1·04, 1·45	Age, sex, energy intake, number of 24-h dietary records, smoking status, educational level, physical activity, BMI, alcohol intake, family history of CVD, baseline prevalent type 2 diabetes, dyslipidaemia, hypertension, hypertriacylglycerolaemia and treatments for these conditions	Good
		665/105 159				CHD risk					1·18	0·93, 1·52		
		892/105 159				CV risk					1·23	1·00, 1·53		
Bonaccio *et al*. (2020)^(35)^	Italy (Moli-sani Study)	1235/22 810	8·3	55	M/F	All-cause mortality	188-item FFQ	Q4 *v*. Q1	>4 servings/d	<2 servings/d	1·15	1·00, 1·34	NA	NA
		NA				CVD mortality					1·50	1·18, 1·92		
		NA				IHD/CV mortality					1·56	1·13, 2·14		

CDS, Cognitive Difficulties Scale; CES-D, Center for Epidemiologic Studies Depression Scale; CV, cerebrovascular; ELSA, Brazilian Longitudinal Study of Adult Health; ENRICA, Study on Nutrition and Cardiovascular risk factors in Spain; F, female; F-up, follow-up; M, male; mPNNS-GS, Modified Programme National Nutrition Santé Guideline Score; NA, not available; NHANES, National Health and Nutrition Examination Survey; RR, risk ratio; TV, television; SUN, University of Navarra Follow-Up Project; WC, waist circumference.

**Fig. 3.**
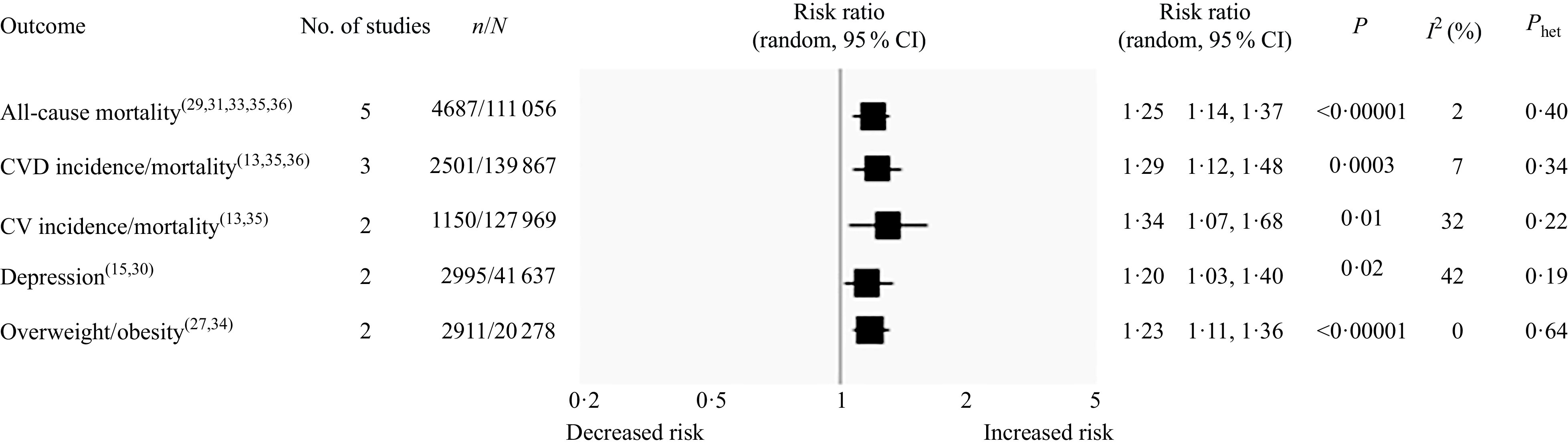
Forest plot of prospective cohort studies investigating the association between ultra-processed foods consumption and different health outcomes. *P* value is for *Z* test of no overall association between exposure and outcome; *P*_het_ is for test of no differences in association measure among studies; *I* ^2^ estimates from heterogeneity rather than sampling error. CV, cerebrovascular.

The results of the pooled analysis for all included studies are shown in Fig. 3. The highest consumption of UPF was found to be associated with an increased risk of all-cause mortality in five studies^(29,31,33,35,36)^ involving 111 056 subjects and 4687 deaths (RR 1·25, 95 % CI 1·14, 1·37; *P* < 0·00001), with no statistical heterogeneity between studies (*I* ^2^ = 2 %; *P* = 0·40). In addition, highest UPF intake showed a significant association with increased risk of CVD incidence and/or mortality in three studies^(13,35,36)^ with 2501 cases (RR 1·29, 95 % CI 1·12, 1·48; *P* = 0·0003; *I* ^2^ = 7 %, *P* = 0·34), cerebrovascular disease incidence and/or mortality in two studies^(13,35)^ with 1150 cases (RR 1·34, 95 % CI 1·07, 1·68; *P* = 0·01; *I* ^2^ = 32 %, *P* = 0·22) and depression in two studies^(15,30)^ with 2995 cases (RR 1·20, 95 % CI 1·03, 1·40; *P* = 0·02; *I* ^2^ = 42 %, *P* = 0·19). The statistically significant association was also found for overweight/obesity in two studies^(27,34)^ with 2911 cases, (RR 1·23, 95 % CI 1·11, 1·36; *P* < 0·0001) and no evidence of heterogeneity between studies (*I* ^2^ = 0 %, *P* = 0·64).

### Sensitivity analysis and publication bias

A leave-one-out sensitivity analysis was performed by iteratively removing one study at a time to confirm that our results were not determined by a single study. There were few changes in the quantitative measurements of OR, RR and the 95 % CI, without any study affecting the results for almost all of the outcomes investigated. The only exceptions were found in cross-sectional study analyses for the metabolic syndrome, low HDL-cholesterol levels and hyperglycaemia. For the metabolic syndrome and low HDL-cholesterol levels, the removal of the study by Lavigne-Robichaud *et al*.^(24)^ changed the relative effect from significant (in the main analysis) to non-significant in the sensitivity analysis (OR 1·11, 95 % CI 0·26, 4·65; *P* = 0·89 and OR 1·82, 95 % CI 0·52, 6·42; *P* = 0·35). Conversely, for hyperglycaemia, the removal of the study by Nasreddine *et al*.^(12)^ changed the relative effect from non-significant in the main analysis to significant in the sensitivity analysis (OR 1·76, 95 % CI 1·04, 2·97; *P* = 0·03).

The publication bias was evaluated for all-cause mortality (online Supplementary Fig. S1). The shape of the funnel plot did not show any evident asymmetry, suggesting the absence of possible publication biases.

## Discussion

The present study is the first systematic review with meta-analysis that evaluated all available observational studies that assessed the association between UPF consumption and health status. By comparing the highest *v*. the lowest UPF consumption, the pooled analysis of cross-sectional studies, carried out for each result in a limited number of studies, showed a possible increase in the risk of overweight/obesity, high waist circumference, reduced levels of HDL-cholesterol and the metabolic syndrome. Similarly, for prospective cohort studies, the increased UPF consumption was associated with an increased risk of all-cause mortality in five studies, CVD in three studies, cerebrovascular disease and depression in only two studies, and confirmed the significant association found for overweight/obesity, but only in two studies. These results, although reporting interesting and useful data to formulate a hypothesis, must be carefully interpreted due to the low number of subjects and studies investigated.

In recent years, the global food system has undergone a profound transformation in terms of technology and food processing. The food profile of the world’s countries has changed significantly in favour of the consumption of highly processed industrial products for reasons of economic convenience, industrial competition and attractiveness to the consumer^(37)^. For all these reasons, the availability and consumption of UPF have increased significantly in all countries, regardless of economic level^(38)^. These foods are defined based on a classification system called *NOVA*, which classifies foods into four groups according to the industrial processing used in their production^(1)^. The *NOVA* classification is a simple classification based on the food technology and does not provide any indications on the nutritional content of the food. According to this classification, UPF are defined as products ‘created mostly or entirely from substances extracted from food or derived from food constituents with little or no intact food’. Since Monteiro coined the term UPF, there have been an increasing number of studies that have associated UPF consumption with negative health outcomes in adult subjects^(3,39)^, including cardiometabolic risk factors^(40)^, CVD^(35)^, cancer^(16)^ and many other outcomes^(15,25,32)^.

In the present study, we have thoroughly evaluated all the observational studies that investigated the possible association between UPF consumption and health status, and we made a quantitative assessment of the association through the meta-analytical procedure. The number of studies included was limited, especially for individual outcomes, and does not allow us to be sure that the results obtained are completely reliable but is sufficient to hypothesise the nature of the association that must then be tested in intervention studies for validation. The analysis of the ten cross-sectional studies showed an increased risk of overweight/obesity, high waist circumference, reduced HDL-cholesterol levels and the metabolic syndrome but not of the other outcomes such as hypertension, hyperglycaemia or hypertriacylglycerolaemia in adults who consume high levels of UPF compared with those who consume less. The analysis of prospective cohort studies confirmed the significant increase in the risk of overweight/obesity and documented a 29 % increase in the risk of CVD incidence and/or mortality, a 34 % increase in the risk of cerebrovascular disease and a 20 % increase in the risk of depression.

The explanations for the possible harmful effects of UPF on health are different and may lie in the fact that these foods are, *de facto*, indicators of poor food quality, containing high amounts of free or added sugars, fats, low levels of fibre and high energy density^(41)^. These characteristics can reasonably explain the negative effect of these products on cardiovascular and cardiometabolic risk factors, as well as the risk of overweight/obesity. However, beyond the nutritional composition, UPF could also explain their harmful effects through other mechanisms, such as the presence of compounds that are formed during the processing of the food, and therefore more present in UPF. For example, both acrylamide – a contaminant present in heat-treated processed food products – and acrolein – a compound formed during fat heating – have been associated with an increased risk of CVD^(42,43)^. In addition, bisphenol A – an industrial chemical used in some UPF plastic packaging – has been found associated with an increased risk of cardiometabolic disorders^(44)^. Although bisphenol A is banned for use in food packaging in many countries, it has now been replaced by other components such as bisphenol S, which also has endocrine-disrupting properties, and is suspected to be absorbed more orally than bisphenol A^(45)^. Recent studies have confirmed that UPF consumption is associated with increased exposure to endocrine-disrupting chemicals and phthalates used in industrial plastic packaging^(45,46)^. Another possible explanation for the harmful effects of UPF on health status is related to their organoleptic characteristics, which have led to an increase in the eating rate and delayed satiety signalling, leading to higher overall food intake. Hall *et al*. recently^(47)^ conducted a randomised controlled trial on twenty weight-stable adults who were randomised to receive either ultra-processed or unprocessed *ad libitum* diets for 2 weeks. At the end of the study, a significant increase in body weight was reported along with an overall increase in energy intake only after the UPF-rich diet. In addition, it was also hypothesised that UPF can adversely affect health by modifying the gut microbiome in such a way that it disturbs the energy balance and promotes the selection of microbes that promote inflammation-related diseases such as CVD and metabolic diseases and even depression^(48,49)^.

The present study has several limitations that should be addressed. First, the included studies evaluated UPF consumption through self-reported tools (FFQ, food records and 24-h recalls), which are generally accepted, but which are susceptible to recall bias, and which are not specifically designed to collect UPF data as described by the *NOVA* classification. This may result in an over- or underestimation of the UPF intake level. Indeed, the application of the National Institutes of Health study quality assessment tool suggested that the methodological quality of all the cross-sectional studies included was fair or poor, mainly due to the lack of details on the validity and reliability of the questionnaires used to assess UPF consumption. Secondly, the overall analyses for each different outcome were carried out in a limited number of studies, thus reducing the statistical power of the analysis. Third, only a limited number of studies included total energy intake as a confounding variable in the multivariable models, thus introducing a possible limitation in the interpretation of the results. However, it should be noted that total energy intake can also be part of the causal pathway of UPF intake; therefore, this aspect is not necessarily a study limitation. In addition, it is well known that unhealthy eating habits (i.e. high consumption of UPF) are commonly associated with other unhealthy lifestyle behaviours, such as sedentary habits, which in turn are associated with adverse health outcomes. Thus, the results of the present meta-analysis should be interpreted with caution, since not all the included studies considered unhealthy lifestyle behaviours as confounding factors in the multivariable models. On the other hand, the study presents also some strengths such as a rigorous search and selection strategy that identified all available cross-sectional and prospective cohort studies examining the relationship between UPF consumption and health status, and the fact that all but one of the included cohort studies had good methodological quality, with an adequate follow-up, and high participation rates.

In conclusion, we reported for the first time in a systematic review with meta-analysis the possible association between high UPF consumption, worse cardiometabolic risk profile (reported mainly by an increased risk of overweight/obesity, elevated waist circumference, reduced HDL-cholesterol levels and increased risk of the metabolic syndrome), and greater risk of all-cause mortality, CVD, cerebrovascular disease and depression. The available literature still has several limitations and the methods used to classify these foods need careful review, so reducing the applicability and transferability of these results to the general population. However, these findings have important public health implications, especially for food policymakers who should discourage the consumption of UPF and promote fresh and minimally processed foods to improve health status.

## Supplementary material

For supplementary material accompanying this paper visit http://dx.doi.org/10.1017/S0007114520002688. click here to view supplementary material
