# Supplementary material for: Consumption of ultra-processed foods and health status: a systematic review and meta-analysis
Source: Br J Nutr. 2020 Aug 14;125(3):308–18. doi: 10.1017/S0007114520002688 (PMC7844609; doi:10.1017/S0007114520002688)
Supplement: Supplementary file 1 [file S0007114520002688sup.zip › S0007114520002688sup002.docx]

**Supplementary Table 1.** PECOS criteria used in the present meta-analysis

| **Parameter** | **Description** |
| --- | --- |
| **Population** | Inclusion criteria: Clinically healthy subjects aged ≥18 years, all ethnicities  Exclusion criteria: Subjects aged <18 years, pregnant women |
| **Exposure** | High consumption of ultra-processed foods as defined by the NOVA Food Classification System |
| **Comparison** | Low consumption of ultra-processed foods as defined by the NOVA Food Classification System |
| **Outcome** | Any health indicator |
| **Study design** | Inclusion criteria: Cross-sectional studies, prospective cohort studies  Exclusion criteria: Review articles, letters to the editor, comments, case reports, case-control studies, randomized controlled trials |

**Supplementary Table 2.** **NIH Quality Assessment Tool**

| **Author, year** | **Q1** | **Q2** | **Q3** | **Q4** | **Q5** | **Q6** | **Q7** | **Q8** | **Q9** | **Q10** | **Q11** | **Q12** | **Q13** | **Q14** | **Quality rating** |
| --- | --- | --- | --- | --- | --- | --- | --- | --- | --- | --- | --- | --- | --- | --- | --- |
| **CROSS-SECTIONAL STUDIES** |  |  |  |  |  |  |  |  |  |  |  |  |  |  |  |
| **Louzada et al., 2015 ^6^** | Yes | Yes | No | Yes | No | No | No | Yes | Yes | No | Yes | NR | NA | Yes | **Fair** |
| **Juul et al., 2018 ^7^** | Yes | Yes | No | Yes | No | No | No | Yes | Yes | No | Yes | NR | NA | Yes | **Fair** |
| **Lavigne-Robichaud et al., 2018 ^24^** | Yes | Yes | Yes | Yes | No | No | No | Yes | No | No | Yes | NR | NA | Yes | **Poor** |
| **Nasreddine et al., 2018 ^12^** | Yes | Yes | Yes | Yes | Yes | No | No | Yes | No | No | Yes | NR | NA | Yes | **Poor** |
| **Schnabel et al., 2018 ^25^** | Yes | Yes | No | Yes | No | No | No | Yes | Yes | Yes | Yes | NR | NA | Yes | **Fair** |
| **Silva et al., 2018 ^8^** | Yes | Yes | Yes | Yes | No | No | No | Yes | Yes | No | Yes | NR | NA | Yes | **Fair** |
| **Lopes et al., 2019 ^23^** | Yes | Yes | Yes | Yes | No | No | No | Yes | Yes | No | Yes | NR | NA | Yes | **Poor** |
| **Martínez Steele et al., 2019 ^10^** | Yes | Yes | No | Yes | No | No | No | Yes | Yes | No | Yes | NR | NA | Yes | **Fair** |
| **Nardocci et al., 2019 ^9^** | Yes | Yes | Yes | Yes | No | No | No | Yes | No | No | No | NR | NA | Yes | **Poor** |
| **Rauber et al., 2020 ^26^** | Yes | Yes | No | Yes | No | No | No | Yes | Yes | No | Yes | NR | NA | Yes | **Fair** |
| **PROSPECTIVE COHORT STUDIES** |  |  |  |  |  |  |  |  |  |  |  |  |  |  |  |
| **Mendonça et al., 2016 ^27^** | Yes | Yes | No | Yes | No | Yes | Yes | Yes | Yes | No | Yes | NR | Yes | Yes | **Good** |
| **Mendonça et al., 2017 ^28^** | Yes | Yes | Yes | Yes | No | Yes | Yes | Yes | Yes | No | Yes | NR | Yes | Yes | **Good** |
| **Fiolet et al., 2018 ^16^** | Yes | Yes | NR | Yes | No | Yes | Yes | Yes | Yes | Yes | Yes | NR | NR | Yes | **Good** |
| **Adjibade et al., 2019 ^15^** | Yes | Yes | Yes | Yes | No | Yes | Yes | Yes | Yes | Yes | Yes | NR | NR | Yes | **Good** |
| **Blanco-Rojo et al., 2019 ^29^** | Yes | Yes | Yes | Yes | No | Yes | Yes | Yes | Yes | No | Yes | NR | NR | Yes | **Good** |
| **Canhada et al., 2019 ^34^** | Yes | Yes | Yes | Yes | No | Yes | Yes | Yes | Yes | No | Yes | NR | NR | Yes | **Good** |
| **Gómez-Donoso et al., 2019 ^30^** | Yes | Yes | Yes | Yes | No | Yes | Yes | Yes | Yes | Yes | Yes | NR | Yes | Yes | **Good** |
| **Kim et al., 2019 ^36^** | Yes | Yes | Yes | Yes | No | Yes | Yes | Yes | Yes | No | Yes | NR | Yes | Yes | **Good** |
| **Rico-Campà et al., 2019 ^31^** | Yes | Yes | Yes | Yes | No | Yes | Yes | Yes | Yes | Yes | Yes | NR | Yes | Yes | **Good** |
| **Sandoval-Insausti et al., 2019 ^32^** | Yes | Yes | Yes | Yes | No | Yes | Yes | Yes | Yes | No | No | NR | Yes | Yes | **Fair** |
| **Schnabel et al., 2019 ^33^** | Yes | Yes | No | Yes | No | Yes | Yes | Yes | Yes | No | Yes | NR | Yes | Yes | **Good** |
| **Srour et al., 2019a ^13^** | Yes | Yes | Yes | Yes | No | Yes | Yes | Yes | Yes | Yes | Yes | NR | Yes | Yes | **Good** |

Q1 = Was the research question or objective in this paper clearly stated?; Q2 = Was the study population clearly specified and defined?; Q3 = Was the participation rate of eligible persons at least 50%?; Q4 = Were all the subjects selected or recruited from the same or similar populations (including the same time period)? Were inclusion and exclusion criteria for being in the study prespecified and applied uniformly to all participants?; Q5 = Was a sample size justification, power description, or variance and effect estimates provided?; Q6 = For the analyses in this paper, were the exposure(s) of interest measured prior to the outcome(s) being measured?; Q7 = Was the timeframe sufficient so that one could reasonably expect to see an association between exposure and outcome if it existed?; Q8 = For exposures that can vary in amount or level, did the study examine different levels of the exposure as related to the outcome (e.g., categories of exposure, or exposure measured as continuous variable)?; Q9 = Were the exposure measures (independent variables) clearly defined, valid, reliable, and implemented consistently across all study participants?; Q10 = Was the exposure(s) assessed more than once over time?; Q11 = Were the outcome measures (dependent variables) clearly defined, valid, reliable, and implemented consistently across all study participants?; Q12 = Were the outcome assessors blinded to the exposure status of participants?; Q13 = Was loss to follow-up after baseline 20% or less?; Q14 = Were key potential confounding variables measured and adjusted statistically for their impact on the relationship between exposure(s) and outcome(s)?; NA = Not Applicable; NR = Not Reported
