# Supplementary figures and images for: Consumption of ultra-processed foods and health status: a systematic review and meta-analysis
Source: Br J Nutr. 2020 Aug 14;125(3):308–18. doi: 10.1017/S0007114520002688 (PMC7844609; doi:10.1017/S0007114520002688)

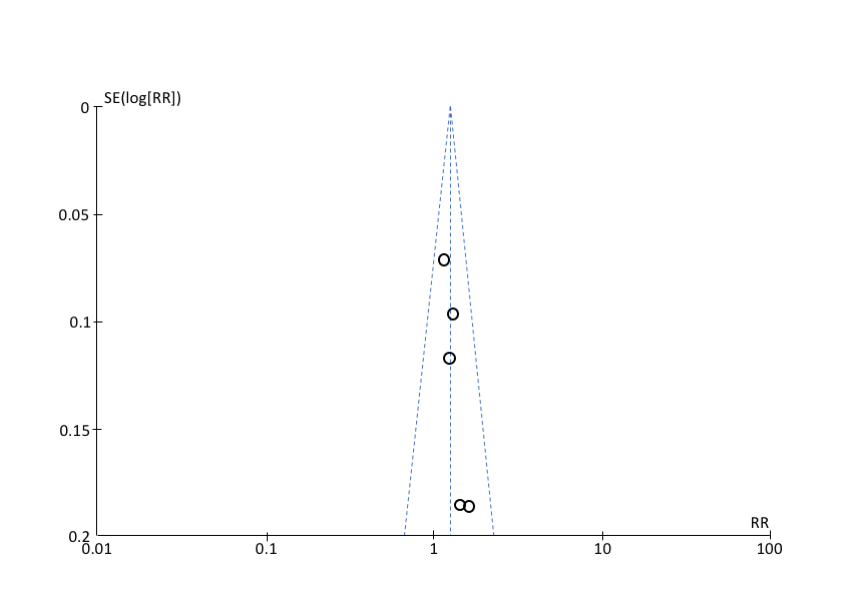

Supplement: Supplementary file 1 [file S0007114520002688sup.zip › S0007114520002688sup001.tiff]
